# Analysis on the Radial Vibration of Longitudinally Polarized Radial Composite Tubular Transducer

**DOI:** 10.3390/s20174785

**Published:** 2020-08-25

**Authors:** Xiaoyu Wang, Shuyu Lin

**Affiliations:** Shaanxi Key Laboratory of Ultrasonics, Institute of Applied Acoustics, Shaanxi Normal University, Xi’an 710119, China; wangxiaoyu1202@snnu.edu.cn

**Keywords:** longitudinally polarized, tubular transducer, equivalent circuit, vibration characteristics

## Abstract

The radial vibration of a radial composite tubular transducer with a large radiation range and power capacity is studied. The transducer is composed of a longitudinally polarized piezoelectric ceramic tube and a coaxial outer metal tube. Assuming that the longitudinal length is much larger than the radius, the electromechanical equivalent circuits of the radial vibration of a piezoelectric ceramic long tube and a metal long tube are derived and obtained for the first time following the plane strain theory. As per the condition of the continuous forces and displacements of two contact surfaces, the electromechanical equivalent circuit of the tubular transducer is obtained. The radial resonance/anti-resonance frequency equation and the expression of the effective electromechanical coupling coefficient are obtained. Then, the effects of the radial geometry dimension of the transducer on the vibration characteristics are analyzed. The theoretical resonance frequencies, anti-resonance frequencies, and the effective electromechanical coupling coefficients at the fundamental mode and the second mode are in good agreement with the finite element analysis (FEA) results. The study shows that when the overall size of the transducer is unchanged, as the proportion of piezoelectric ceramic increases, the radial resonance/anti-resonance frequency and the effective electromechanical coupling coefficient of the transducer at the fundamental mode and the second mode have certain characteristics. The radial composite tubular transducer is expected to be used in high-power ultrasonic wastewater treatment, ultrasonic degradation, and underwater acoustics, as well as other high-power ultrasonic fields.

## 1. Introduction

Longitudinal sandwich piezoelectric ceramic ultrasonic transducers, which are also called Langevin piezoelectric composite ultrasonic transducers, are widely used in high-power ultrasound [1,2,3,4]. The traditional sandwich ultrasonic transducers have the advantages of a simple structure, adjustable performance, and high electro-acoustic efficiency. However, their radial dimension is required to be much smaller than 1/4 wavelength so that the power capacity, radiation area, and output power of the transducers are all limited. Piezoelectric ultrasonic vibration systems with radial composite structures are widely used in ultrasonic cleaning, ultrasonic degradation, and underwater acoustics because of their two-dimensional radiation surface, high power capacity, large output power, and uniform directivity [5,6,7,8].

Until now, the research on radial composite ultrasonic transducers has been relatively comprehensive. According to the ratio of the longitudinal length of the radius, the research can be divided into the following three categories: When the ratio is small, the model of the piezoelectric element is mainly a thin solid disk or a hollow ring. Iula et al. analyzed the radial symmetry mode of the thin piezoelectric ring [9,10]. Ganilova and Guo analyzed the radial vibration of the disc-type transducer using an analytical method [11] and a finite element method [12], respectively. Lin et al. deduced the electromechanical-equivalent circuits of longitudinally polarized piezoelectric ceramic discs and rings [13,14,15]. In the derivation, based on the plane stress theory about mechanics, the vibration of the transducers is considered to be a uniform axisymmetric radial vibration. When the longitudinal length is comparable to the radius, the piezoelectric element is mainly a hollow cylinder. In this case, the vibration of the transducer is such a complex coupled vibration that there are only a few theories about it [16,17]. In general, the finite element analysis method is used to analyze the coupled vibration of the transducer [18,19,20]. When the longitudinal length is much larger than the radius, the piezoelectric element is usually a hollow long tube. Lin et al. deduced the electromechanical equivalent circuit of the radial vibration of the radially polarized piezoelectric ceramic long tube with an arbitrary wall thickness [21]. In the analysis, the vibration theory of the long tube is regarded as a plane strain problem, which is completely different from the two categories mentioned above.

Generally speaking, there are many methods to analyze the vibrations of piezoelectric ceramic vibrators, and the most widely used is the equivalent circuit method [22,23,24,25]. Mason used the electromechanical properties of piezoelectric vibrators to calculate the impedance and resonance frequency [26]. Martin derived the equivalent circuit of the longitudinally polarized thin-walled ferroelectric ceramic tube in a longitudinal vibration [27]. Until now, some basic piezoelectric vibrators have had corresponding electromechanical equivalent circuits [28,29,30,31].

Along with the development of ultrasonic technology, the optimization of high-power ultrasonic transducers is mainly focused on two aspects. On the one hand, the power capacity of the piezoelectric vibrator is expected to improve in order to increase the power of the whole transducer. On the other hand, the tool heads of the different models are designed to increase the displacement amplitude of the radiated end face of the transducer. In order to enlarge the radiation area, improve the power capacity, and improve the equivalent circuit theory of the piezoelectric vibrator, the radial vibration of a high-power longitudinally polarized radial composite tubular transducer is studied. In the analysis, the plane strain theory is considered, and the vibration of the transducer can be regarded as an axisymmetric radial vibration. Based on the theoretical analysis, the electromechanical equivalent circuit of the radial vibration of the transducer is derived. In order to verify the correctness of the analytical theory, finite element analysis (FEA) is employed to simulate the vibrational modes of the transducer.

## 2. Theoretical Analysis of Radial Composite Tubular Transducer

**Figure 1:**
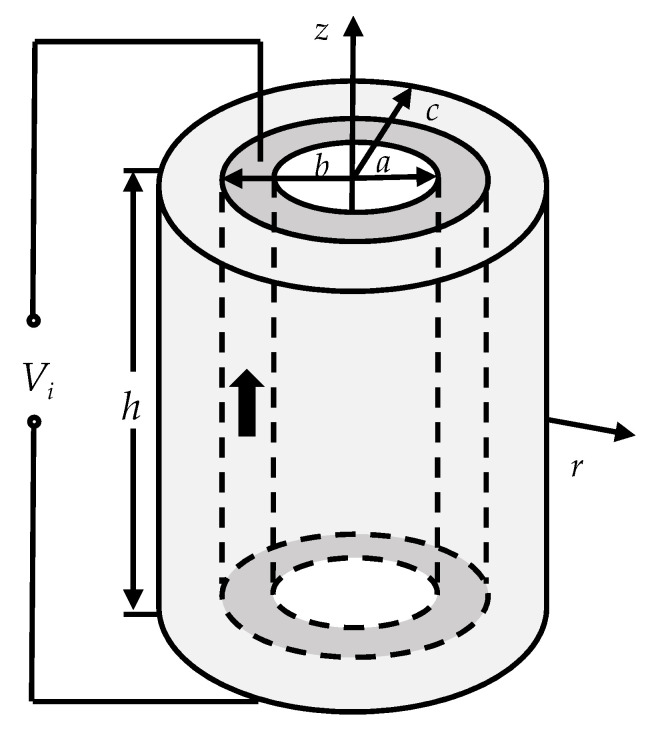
Radial composite tubular transducer.

Figure 1 illustrates a radial composite tubular transducer. In the figure, a cylindrical coordinate system is established, with the central position of the transducer as the origin, and *r* and *z* as the radial and axial directions, respectively. The transducer is composed of a longitudinally polarized piezoelectric ceramic tube and a coaxial outer metal tube in the radial direction. The radius of the transducer is *a*, *b*, and *c* from inside to outside; the height of the transducer is *h*, and *h* is much larger than *c*. The direction of the applied electric field of the piezoelectric ceramic tube is parallel to its polarization direction.

### 2.1. Equivalent Circuit of the Piezoelectric Ceramic Tube

In the cylindrical coordinate system, the motion equation of the radial vibration of the piezoelectric ceramic tube [16] is

(1) $$\rho_{r}\frac{\partial^{2}\xi_{r}}{\partial t^{2}}=\frac{\partial T_{r}}{\partial r}+\frac{1}{r}\frac{\partial T_{r\theta }}{\partial \theta }+\frac{\partial T_{rz}}{\partial z}+\frac{T_{r}-T_{\theta }}{r}$$

where $T_{i}$ (*i* = *r*, *θ*, *rθ*, *rz*) are the stress components of the piezoelectric ceramic tube, $\xi_{r}$ and $\xi_{\theta }$ are the radial and circumferential displacement components of radial vibration of the piezoelectric ceramic tube, and $\rho_{r}$ is the density of the piezoelectric material. The relationships between the strain components and the displacement components are

(2) $$S_{r}=\frac{\partial \xi_{r}}{\partial r}$$

(3) $$S_{\theta }=\frac{1}{r}\frac{\partial \xi_{\theta }}{\partial \theta }+\frac{\xi_{r}}{r}$$

where $S_{i}$ (*i* = *r*, *θ*) are the radial and circumferential strain components. Because the vibration of the transducer is an axisymmetric radial vibration, $T_{r\theta }=T_{rz}=0$, $\xi_{\theta }$ = 0. Equations (1) and (3) can be simplified as

(4) $$\rho_{r}\frac{\partial^{2}\xi_{r}}{\partial t^{2}}=\frac{\partial T_{r}}{\partial r}+\frac{T_{r}-T_{\theta }}{r}$$

(5) $$S_{\theta }=\frac{\xi_{r}}{r}$$

In the cylindrical coordinate system, the linear piezoelectric constitutive equations of a longitudinally polarized piezoelectric ceramic tube [32] are as follows:

(6) $$S_{r}=s_{11}^{D}T_{r}+s_{12}^{D}T_{\theta }+s_{13}^{D}T_{z}+g_{31}D_{3}$$

(7) $$S_{\theta }=s_{12}^{D}T_{r}+s_{11}^{D}T_{\theta }+s_{13}^{D}T_{z}+g_{31}D_{3}$$

(8) $$S_{z}=s_{13}^{D}T_{r}+s_{13}^{D}T_{\theta }+s_{33}^{D}T_{z}+g_{31}D_{3}$$

(9) $$E_{3}=-g_{31}T_{r}-g_{31}T_{\theta }-g_{33}T_{z}+\beta_{33}^{T}D_{3}$$

where $S_{z}$ is the axial strain component, $S_{\beta \gamma }^{D}$ (β = 1, 3, γ = 1, 2, 3) are the elastic compliance constants, $g_{31}$ and $g_{33}$ are piezoelectric constants, and $\beta_{33}^{T}$ is the free dielectric isolation rate. $E_{3}$ and $D_{3}$ are the applied excitation electric field and the electric displacement vector, respectively. When the longitudinal length, *h*, of the tube is much larger than the radius, *c*, the plane strain theory about mechanics can be applied to analyze the vibration of the transducer, so $S_{z}=0$. By combining Equations (8) and (9) with $S_{z}=0$, the following expressions can be obtained

(10) $$T_{z}=\frac{-g_{33}}{\beta_{33}^{T}s_{33}^{D}+g_{33}{}^{2}}E_{3}-\frac{\beta_{33}^{T}s_{33}^{D}+g_{31}g_{33}}{\beta_{33}^{T}s_{33}^{D}+g_{33}{}^{2}}T_{r}-\frac{\beta_{33}^{T}s_{33}^{D}+g_{31}g_{33}}{\beta_{33}^{T}s_{33}^{D}+g_{33}{}^{2}}T_{\theta }$$

(11) $$D_{3}=\frac{s_{33}^{D}}{\beta_{33}^{T}s_{33}^{D}+g_{33}{}^{2}}E_{3}-\frac{s_{13}^{D}g_{33}-g_{31}s_{33}^{D}}{\beta_{33}^{T}s_{33}^{D}+g_{33}{}^{2}}T_{r}-\frac{s_{13}^{D}g_{33}-g_{31}s_{33}^{D}}{\beta_{33}^{T}s_{33}^{D}+g_{33}{}^{2}}T_{\theta }$$

Substituting Equations (10) and (11) into Equations (6) and (7), the expressions of the radial strain component, $S_{r}$, and circumferential strain component, $S_{\theta }$, can be simplified as

(12) $$S_{r}=A_{1}T_{r}+A_{2}T_{\theta }+A_{3}E_{3}$$

(13) $$S_{\theta }=A_{2}T_{r}+A_{1}T_{\theta }+A_{3}E_{3}$$

where *A*_1_, *A*_2_, and *A*_3_ are constants, which are defined as $A_{1}=s_{11}^{D}-\left(\beta_{33}^{T}s_{13}^{D}{}^{2}+2g_{31}g_{33}s_{13}^{D}-g_{31}^{2}s_{33}^{D}\right)/\left(\beta_{33}^{T}s_{33}^{D}+g_{33}^{2}\right)$, $A_{2}=s_{12}^{D}-\left(\beta_{33}^{T}s_{13}^{D}{}^{2}+2g_{31}g_{33}s_{13}^{D}-g_{31}^{2}s_{33}^{D}\right)/\left(\beta_{33}^{T}s_{33}^{D}+g_{33}^{2}\right)$, and $A_{3}=\left(g_{31}s_{33}^{D}-g_{33}s_{13}^{D}\right)/\left(\beta_{33}^{T}s_{33}^{D}+g_{33}^{2}\right)$.

From Equations (12) and (13), the radial stress component and the relationships between the radial and circumferential stress components can be obtained as

(14) $$T_{r}=\left(\frac{S_{r}+S_{\theta }-2A_{3}E_{3}}{A_{1}+A_{2}}+\frac{S_{r}+S_{\theta }}{A_{1}-A_{2}}\right)/2$$

(15) $$T_{r}-T_{\theta }=\frac{S_{r}-S_{\theta }}{A_{1}-A_{2}}$$

(16) $$T_{r}+T_{\theta }=\frac{S_{r}+S_{\theta }-2A_{3}E_{3}}{A_{1}+A_{2}}$$

Substituting Equations (2) and (5) into Equations (14) and (15), and then substituting the obtained results into Equation (4), the wave equation of the piezoelectric ceramic tube on the radial vibration can be obtained. The simple harmonic motion is considered as $\xi_{r}=\xi_{r0}\exp(j\omega t)$. The wave equation can be further written as

(17) $$\frac{d^{2}\xi_{r0}}{dr^{2}}+\frac{1}{r}\frac{d\xi_{r0}}{dr}+(k_{r}^{2}-\frac{1}{r^{2}})\xi_{r0}=0$$

where $k_{r}=\omega /c_{r}$ is the wave number of the radial vibration, $c_{r}=\sqrt{A_{1}/\left(\rho_{r}(A_{1}^{2}-A_{2}^{2})\right)}$ is the speed of sound, and ω is the angular frequency. The displacement expression of the radial vibration can be obtained from Equation (17) as

(18) $$\xi_{r}=\left[B_{1}J_{1}(k_{r}r)+B_{2}Y_{1}(k_{r}r)\right]\exp(j\omega t)$$

where *B*_1_ and *B*_2_ are the constants that are determined by the boundary conditions. $J_{1}(k_{r}r)$ and $Y_{1}(k_{r}r)$ are the Bessel functions of the first and second kinds of order one, respectively. From Equation (18), the radial velocity amplitude can be obtained as

(19) $$v_{r}=j\omega \left[B_{1}J_{1}(k_{r}r)+B_{2}Y_{1}(k_{r}r)\right]\exp(j\omega t)$$

Substituting boundary conditions ${v_{r}|}_{r=a}=v_{a}$, ${v_{r}|}_{r=b}=-v_{b}$ into Equation (19), constants *B*_1_ and *B*_2_ can be obtained as

(20) $$B_{1}=-\frac{1}{j\omega }\frac{v_{a}Y_{1}(k_{r}b)+v_{b}Y_{1}(k_{r}a)}{Y_{1}(k_{r}a)J_{1}(k_{r}b)-Y_{1}(k_{r}b)J_{1}(k_{r}a)}$$

(21) $$B_{2}=\frac{1}{j\omega }\frac{v_{a}J_{1}(k_{r}b)+v_{b}J_{1}(k_{r}a)}{Y_{1}(k_{r}a)J_{1}(k_{r}b)-Y_{1}(k_{r}b)J_{1}(k_{r}a)}$$

Substituting Equation (18) into Equations (2) and (5), and then substituting the result into Equation (14) and combining it with Equations (20) and (21), the radial stress component can be further written as

(22) $$T_{r}=\frac{1}{j\omega \Delta 1(A_{1}^{2}-A_{2}^{2})}\left\{\begin{array}{l} A_{1}k_{r}\left[Y_{0}(k_{r}r)J_{1}(k_{r}b)-J_{0}(k_{r}r)Y_{1}(k_{r}b)\right]v_{b}\\ +\frac{A_{1}+A_{2}}{r}\left[J_{1}(k_{r}r)Y_{1}(k_{r}b)-Y_{1}(k_{r}r)J_{1}(k_{r}b)\right]v_{b}\\ +A_{1}k_{r}\left[Y_{0}(k_{r}r)J_{1}(k_{r}a)-J_{0}(k_{r}r)Y_{1}(k_{r}a)\right]v_{c}\\ +\frac{A_{1}+A_{2}}{r}\left[J_{1}(k_{r}r)Y_{1}(k_{r}a)-Y_{1}(k_{r}r)J_{1}(k_{r}a)\right]v_{c} \end{array}\right\}-\frac{A_{3}E_{3}}{A_{1}+A_{2}}$$

where $\Delta 1{=Y}_{1}(k_{r}a)J_{1}(krb)-Y_{1}(k_{r}b)J_{1}(k_{r}a)$. Substituting the boundary conditions of the external forces $F_{a}={-T_{r}|}_{r=a}S_{1}$ and $F_{b}={-T_{r}|}_{r=b}S_{2}$ into Equation (22) and applying the relationships $F_{a}^{\prime }=n_{1}F_{a}$, $F_{b}^{\prime }=n_{2}F_{b}$, $v_{a}^{\prime }=v_{a}/n_{1}$, $v_{b}^{\prime }=v_{b}/n_{2}$, $n_{1}=\pi k_{r}b/2$, and $n_{2}=\pi k_{r}c/2$, the expressions of the external forces can be obtained as

(23) $$\begin{array}{l} F_{a}^{\prime }=\frac{{\left(\pi k_{r}b\right)}^{2}Z_{1}}{4j}\left[\frac{J_{0}(k_{r}a)Y_{1}(k_{r}b)-Y_{0}(k_{r}a)J_{1}(k_{r}b)}{\Delta 1}+\frac{A_{1}+A_{2}}{k_{r}aA_{1}}\right]v_{a}^{\prime }\\ +j\frac{\pi k_{r}bZ_{1}}{2\Delta 1}v_{b}^{\prime }+\frac{\pi^{2}k_{r}abA_{3}}{A_{1}+A_{2}}V_{i} \end{array}$$

(24) $$\begin{array}{l} F_{b}^{\prime }=\frac{{\left(\pi k_{r}a\right)}^{2}Z_{2}}{4j}\left[\frac{J_{0}(k_{r}b)Y_{1}(k_{r}a)-Y_{0}(k_{r}b)J_{1}(k_{r}a)}{\Delta 1}-\frac{A_{1}+A_{2}}{k_{r}bA_{1}}\right]v_{b}^{\prime }\\ +j\frac{\pi k_{r}aZ_{2}}{2\Delta 1}v_{a}^{\prime }+\frac{\pi^{2}k_{r}abA_{3}}{A_{1}+A_{2}}V_{i} \end{array}$$

where $Z_{1}=\rho_{r}c_{r}S_{1}$, $Z_{2}=\rho_{r}c_{r}S_{2}$, $S_{1}=2\pi ah$, and $S_{2}=2\pi bh$, and $V_{i}=E_{3}h$ is the applied voltage whose direction is parallel to the polarization direction. Equations (22) and (23) can also be rewritten as

(25) $$F_{a}^{\prime }=(Z_{p11}+Z_{p13})v_{a}^{\prime }+Z_{p13}v_{b}^{\prime }+N_{31}V_{i}$$

(26) $$F_{b}^{\prime }=(Z_{p12}+Z_{p13})v_{a}^{\prime }+Z_{p13}v_{b}^{\prime }+N_{31}V_{i}$$

where *Z_p_*_11_, *Z_p_*_12_, and *Z_p_*_13_ are the impedances and *N*_31_ is the electromechanical conversion coefficient of the piezoelectric ceramic tube, whose expressions are

(27) $$Z_{p11}=\frac{{\left(\pi k_{r}b\right)}^{2}Z_{1}}{4j}\left[\frac{J_{0}(k_{r}a)Y_{1}(k_{r}b)-Y_{0}(k_{r}a)J_{1}(k_{r}b)}{\Delta 1}+\frac{A_{1}+A_{2}}{k_{r}aA_{1}}\right]-j\frac{\pi k_{r}bZ_{1}}{2\Delta 1}$$

(28) $$Z_{p12}=\frac{{\left(\pi k_{r}a\right)}^{2}Z_{2}}{4j}\left[\frac{J_{0}(k_{r}b)Y_{1}(k_{r}a)-Y_{0}(k_{r}b)J_{1}(k_{r}a)}{\Delta 1}-\frac{A_{1}+A_{2}}{k_{r}bA_{1}}\right]-j\frac{\pi k_{r}aZ_{2}}{2\Delta 1}$$

(29) $$Z_{p13}=j\frac{\pi k_{r}bZ_{1}}{2\Delta 1}=j\frac{\pi k_{r}aZ_{2}}{2\Delta 1}$$

(30) $$N_{31}=\frac{\pi^{2}k_{r}abA_{3}}{A_{1}+A_{2}}$$

The current flowing into the slender tube is $I_{31}=dQ/dt=j\omega Q$, where $Q=2\pi \int_{a}^{b}D_{3}rdr$ is the surface charge. From Equations (8) and (9), the expression of *D*_3_ can be written as

(31) $$D_{3}=\frac{s_{33}^{D}}{\beta_{33}^{T}s_{33}^{D}+g_{33}^{2}}E_{3}-\frac{s_{13}^{D}g_{33}-g_{31}s_{33}^{D}}{\beta_{33}^{T}s_{33}^{D}+g_{33}^{2}}T_{r}-\frac{s_{13}^{D}g_{33}-g_{31}s_{33}^{D}}{\beta_{33}^{T}s_{33}^{D}+g_{33}^{2}}T_{\theta }$$

Substituting Equation (16) into Equation (11), the expression of *D*_3_ can be further written as

(32) $$D_{3}=\left(\frac{s_{33}^{D}}{\beta_{33}^{T}s_{33}^{D}+g_{33}^{2}}-\frac{2A_{3}^{2}}{A_{1}+A_{2}}\right)E_{3}+\frac{A_{3}}{A_{1}+A_{2}}(S_{r}+S_{\theta })$$

**Figure 2:**
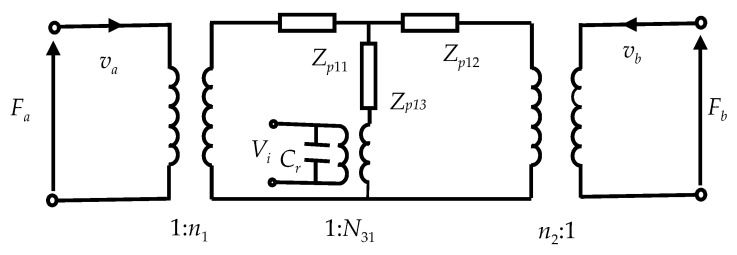
Electromechanical equivalent circuit of the piezoelectric ceramic tube on the radial vibration.

From Equation (32), the current, *I*_31_, can be obtained as

(33) $$I_{31}=j\omega C_{r}V_{i}-N_{31}(v_{a}^{\prime }+v_{b}^{\prime })$$

where $C_{r}=\left(S/h\right)\left[s_{33}^{D}/\left(\beta_{33}^{T}s_{33}^{D}+g_{33}^{2}\right)-2A_{3}^{2}/\left(A_{1}+A_{2}\right)\right]$ is the clamping capacitance in the radial vibration and $S=\pi (b^{2}-a^{2})$ is the cross-sectional area of the tube. According to Equations (25), (26) and (33), the electromechanical equivalent circuit of the piezoelectric ceramic tube on the radial vibration can be obtained as shown in Figure 2.

### 2.2. Electromechanical Equivalent Circuit of the Metal Tube

In the cylindrical coordinate system, according to the generalized Hooke’s law, the relationships between the stresses and strains of a metal tube [33] are

(34) $$T_{r}^{\prime }=E_{1}S_{r}^{\prime }+\sigma_{1}(T_{\theta }^{\prime }+T_{z}^{\prime })$$

(35) $$T_{\theta }^{\prime }=E_{1}S_{\theta }^{\prime }+\sigma_{1}(T_{r}^{\prime }+T_{z}^{\prime })$$

(36) $$\mathrm{and}\;T_{z}^{\prime }=E_{1}S_{z}^{\prime }+\sigma_{1}(T_{\theta }^{\prime }+T_{r}^{\prime })$$

where $T_{\alpha }^{\prime }\left(\alpha =r,\theta ,z\right)$ are the radial, circumferential, and axial stress components, respectively. $S_{\alpha }^{\prime }\left(\alpha =r,\theta ,z\right)$ are the radial, circumferential, and axial stress components, respectively. $E_{1}$ and $\sigma_{1}$ are the Young’s modulus and Poisson’s ratio, respectively. The wave equation of the radial vibration of the metal tube and the relationships between the strain components and the displacement are

(37) $$\rho_{1}\frac{\partial^{2}\xi_{m}}{\partial t^{2}}=\frac{\partial T_{r}^{\prime }}{\partial r}+\frac{T_{r}^{\prime }-T_{\theta }^{\prime }}{r}$$

(38) $$S_{r}^{\prime }=\frac{\partial \xi_{m}}{\partial r}$$

(39) $$S_{\theta }^{\prime }=\frac{\xi_{m}}{\partial r}$$

where $\rho_{1}$ is the density of the metal tube. $\xi_{m}$ is the displacement component of the radial vibration. According to the plane strain problem, substituting $S_{z}=0$ into Equation (36), the expression of $T_{z}^{\prime }$ can be further written as

(40) $$T_{z}^{\prime }=\sigma_{1}(T_{\theta }^{\prime }+T_{r}^{\prime })$$

Substituting Equation (40) into Equations (34) and (35), $T_{r}^{\prime }$ and $T_{\theta }^{\prime }$ can be further written as the following forms

(41) $$T_{r}^{\prime }=\frac{E_{1}}{\left(1+\sigma_{1})(1-2\sigma_{1}\right)}\left[(1-\sigma_{1})S_{r}^{\prime }+\sigma_{1}S_{\theta }^{\prime }\right]$$

(42) $$T_{\theta }^{\prime }=\frac{E_{1}}{\left(1+\sigma_{1})(1-2\sigma_{1}\right)}\left[\sigma_{1}S_{r}^{\prime }+(1-\sigma_{1})S_{\theta }^{\prime }\right]$$

Substituting Equations (38) and (39) into Equations (41) and (42), and then substituting the results into Equation (37), the wave equation of the radial vibration of the metal tube can be further written as

(43) $$\frac{d^{2}\xi_{m0}}{dr^{2}}+\frac{1}{r}\frac{d\xi_{m0}}{dr}+(k_{r1}^{2}-\frac{1}{r^{2}})\xi_{m0}=0$$

where $\xi_{m}=\xi_{m0}\exp(j\omega t)$, $k_{r1}=\omega /c_{r1}$ is the wave number of the radial vibration, $c_{r1}=\sqrt{\left[E_{1}\left(1-\sigma_{1}\right)\right]/\left[\left(1+\sigma_{1}\right)\left(1-2\sigma_{1}\right)\rho_{1}\right]}$ is the speed of sound of the radial vibration in the metal tube, and ω is the angular frequency. The displacement expression of the radial vibration can be obtained from Equation (43) as

(44) $$\xi_{m}=\left[C_{1}J_{1}(k_{r1}r)+C_{2}Y_{1}(k_{r1}r)\right]\exp(j\omega t)$$

where *C*_1_ and *C*_2_ are the constants determined by the boundary conditions. $J_{1}(k_{r1}r)$ and $Y_{1}(k_{r1}r)$ are the Bessel functions of the first and second kinds of order, respectively. From Equation (44), the radial velocity amplitude can be obtained as

(45) $$v_{m}=j\omega \left[C_{1}J_{1}(k_{r1}r)+C_{2}Y_{1}(k_{r1}r)\right]\exp(j\omega t)$$

Substituting boundary conditions ${v_{r}|}_{r=b}=v_{b}$ and ${v_{r}|}_{r=c}=-v_{c}$ into Equation (45), constants *C*_1_ and *C*_2_ can be obtained as

(46) $$C_{1}=\frac{1}{j\omega }\frac{v_{b}Y_{1}(k_{1}c)+v_{c}Y_{1}(k_{1}b)}{Y_{1}(k_{1}c)J_{1}(k_{1}b)-Y_{1}(k_{r}b)J_{1}(k_{r}c)}$$

(47) $$C_{2}=-\frac{1}{j\omega }\frac{v_{b}J_{1}(k_{r1}c)+v_{c}J_{1}(k_{r1}b)}{Y_{1}(k_{r1}c)J_{1}(k_{r1}b)-Y_{1}(k_{r1}b)J_{1}(k_{r1}c)}$$

Substituting Equation (44) into Equations (38) and (39), and then substituting the results into Equation (41) and combining this with Equations (46) and (47), $T_{r}^{\prime }$ can be further written as

(48) $$\begin{array}{l} T_{r}^{\prime }=\frac{jE_{1}(1-\sigma_{1})}{\left(1+\sigma_{1})(1-2\sigma_{1}\right)}\left[\begin{array}{l} \frac{J_{1}(k_{r1}c)Y_{1}^{\prime }(k_{r1}r)-J_{1}^{\prime }(k_{r1}r)Y_{1}(k_{r1}c)}{\Delta 2\omega }v_{b}\\ +\frac{J_{1}(k_{r1}b)Y_{1}^{\prime }(k_{r1}r)-J_{1}^{\prime }(k_{r1}r)Y_{1}(k_{r1}b)}{\Delta 2\omega }v_{c} \end{array}\right]\\ +\frac{jE_{1}\sigma_{1}}{(1+\sigma_{1})(1-2\sigma_{1})r}\left[\begin{array}{l} \frac{J_{1}(k_{r1}c)Y_{1}(k_{r1}r)-J_{1}(k_{r1}r)Y_{1}(k_{r1}c)}{\Delta 2\omega }v_{b}\\ +\frac{J_{1}(k_{r1}b)Y_{1}(k_{r1}r)-J_{1}(k_{r1}r)Y_{1}(k_{r1}b)}{\Delta 2\omega }v_{c} \end{array}\right] \end{array}$$

where $\Delta 2=J_{1}(k_{r1}b)Y_{1}(k_{r1}c)-J_{1}(k_{r1}c)Y_{1}(k_{r1}b)$. Substituting boundary conditions $F_{b}={-T_{r}|}_{r=b}S_{2}$ and $F_{c}={-T_{r}|}_{r=c}S_{3}$ into Equation (48), the external forces of *F_b_* and *F_c_* can be obtained as

(49) $$F_{b}=\frac{Z_{3}}{jk_{r1}}\left\{[\frac{J_{1}(k_{r1}c)Y_{1}^{\prime }(k_{r1}b)-J_{1}^{\prime }(k_{r1}b)Y_{1}(k_{r1}c)}{\Delta 2}-\frac{\sigma_{1}}{\left(1-\sigma_{1}\right)b}]v_{b}+\frac{2}{\pi b\Delta 2}v_{c}\right\}$$

(50) $$F_{c}=\frac{Z_{4}}{jk_{r1}}\left\{\frac{2}{\pi c\Delta 2}v_{b}+[\frac{J_{1}(k_{r1}b)Y_{1}^{\prime }(k_{r1}c)-J_{1}^{\prime }(k_{r1}c)Y_{1}(k_{r1}b)}{\Delta 2}+\frac{\sigma_{1}}{\left(1-\sigma_{1}\right)b}]v_{c}\right\}$$

where $Z_{3}=\rho_{1}c_{r1}S_{2}$,$Z_{4}=\rho_{1}c_{r1}S_{3}$, $S_{3}=2\pi ch$. Equations (49) and (50) can also be rewritten as

(51) $$F_{b}=(Z_{m11}+Z_{m13})v_{b}+Z_{m13}v_{c}$$

(52) $$F_{c}=Z_{m13}v_{b}+(Z_{m12}+Z_{m13})v_{c}$$

**Figure 3:**
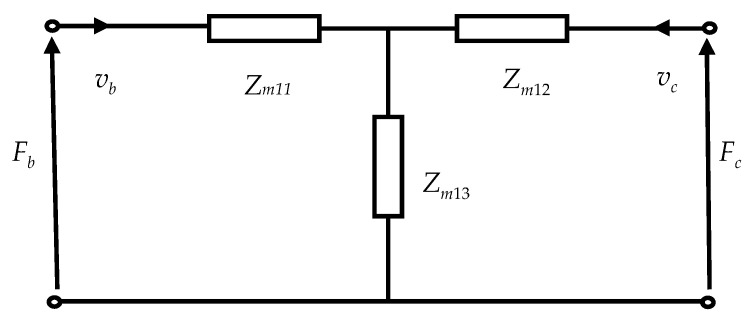
Electromechanical equivalent circuit of the radial vibration of the outer metal tube.

According to Equations (51) and (52), the electromechanical equivalent circuit of the radial vibration of the outer metal tube can be obtained as shown in Figure 3.

In Figure 3, *Z*_*m*11_, *Z*_*m*12_, and *Z*_*m*13_ are the impedances, whose expressions are the following forms:

(53) $$Z_{m11}=\frac{Z_{3}}{jk_{r1}}\left[\frac{J_{1}(k_{r1}c)Y_{1}^{\prime }(k_{r1}b)-J_{1}^{\prime }(k_{r1}b)Y_{1}(k_{r1}c)}{\Delta 2}-\frac{\sigma_{1}}{\left(1-\sigma_{1}\right)b}-\frac{2}{\pi b\Delta 2}\right]$$

(54) $$Z_{m12}=\frac{Z_{4}}{jk_{r1}}\left[\frac{J_{1}(k_{r1}b)Y_{1}^{\prime }(k_{r1}c)-J_{1}^{\prime }(k_{r1}c)Y_{1}(k_{r1}b)}{\Delta 2}+\frac{\sigma_{1}}{\left(1-\sigma_{1}\right)c}-\frac{2}{\pi c\Delta 2}\right]$$

(55) $$Z_{m13}=\frac{2Z_{3}}{j\pi k_{r1}b\Delta 2}=\frac{2Z_{4}}{j\pi k_{r1}c\Delta 2}$$

**Figure 4:**
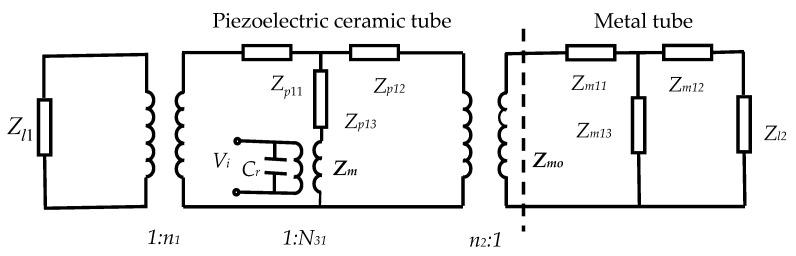
Electromechanical equivalent circuit of a radial composite tubular transducer.

According to the above theoretical analysis, considering the load mechanical resistance and by combining Figure 2 and Figure 3, the electromechanical equivalent circuit of a radial composite tubular transducer can be obtained as shown in Figure 4.

Where *Z*_l1_ and *Z*_l2_ are the load mechanical resistances of the radial composite tubular transducer at the inner and outer surfaces, respectively. The mechanical impedances of the outer metal long tube and the transducer are defined as *Z_mo_* and *Z_m_*, respectively. The input electrical impedance of the transducer is defined as *Z_e_*. The expressions of *Z_mo_*, *Z_m_,* and *Z_e_* can be written as

(56) $$Z_{mo}=Z_{m11}+\frac{(Z_{l2}+Z_{m12})Z_{m13}}{Z_{l2}+Z_{m12}+Z_{m13}}$$

(57) $$Z_{m}=Z_{p13}+\frac{\left(Z_{p11}+n_{1}{}^{2}Z_{l1})(Z_{p12}+n_{2}{}^{2}Z_{mo}\right)}{Z_{p11}+n_{1}{}^{2}Z_{l1}+Z_{p12}+n_{2}{}^{2}Z_{mo}}$$

(58) $$Z_{e}=\frac{Z_{m}}{N_{31}{}^{2}+j\omega C_{r}Z_{m}}$$

Under the no-load state, the resonance frequency equation, anti-resonance frequency equation, and the expression of the effective electromechanical coupling coefficient are

(59) $$Z_{e}=0$$

(60) $$Z_{e}\to \infty$$

(61) $$K_{eff}=\sqrt{1-\left(f_{r}{}^{2}/f_{a}{}^{2}\right)}$$

where *f_r_*, *f_a_,* and *K_eff_* are the resonance frequency, anti-resonance frequency, and the effective electromechanical coupling coefficient, respectively. According to Equations (59)–(61), when the geometric dimensions and the basic material parameters of the transducer are given, the resonance frequency, anti-resonance frequency, and the effective electromechanical coupling coefficient can be obtained.

## 3. Effects of Radial Geometric Dimension and Load Mechanical Resistance of the Radial Composite Tubular Transducer on the Vibration Characteristics

Because Equations (59)–(61) are transcendental equations, Wolfram Mathematica 11.3 was used to calculate the radial resonance frequencies and anti-resonance frequencies of the transducer at the fundamental mode and the second mode. In order to verify the correctness of the theoretical results, the theoretical results were compared with the FEA results, which were obtained by COMSOL Multiphysics 5.3a. PZT-4 was selected as the material of the piezoelectric ceramic tube, which had the following standard material parameters: $\rho_{{}_{r}}=7500\;\mathrm{kg}/m^{3}$, $s_{11}^{D}=10.9\times 10^{-12}\;m^{2}/N$, $s_{12}^{D}=-5.42\times 10^{-12}\;m^{2}/N$, $s_{13}^{D}=-2.1\times 10^{-12}\;m^{2}/N$, $s_{33}^{D}=7.9\times 10^{-12}\;m^{2}/N$, $g_{31}=-11.1\times 10^{-3}\;\mathrm{Vm}/N$, $g_{33}=-26.1\times 10^{-3}\;\mathrm{Vm}/N$, $d_{33}=496\times 10^{-12}\;C/N$, $\varepsilon_{33}^{T}/\varepsilon_{0}=1300$, and $\varepsilon_{0}=8.842\times 10^{-12}\;F/N$. Aluminum was chosen as the material for the outer metal tube, whose standard material parameters were *E*_1_ = $7\times 10^{10}\;\mathrm{Pa}$, $\rho_{{}_{1}}=\;2700\mathrm{kg}/m^{3}$, $\sigma_{1}=0.33$.

**Figure 5:**
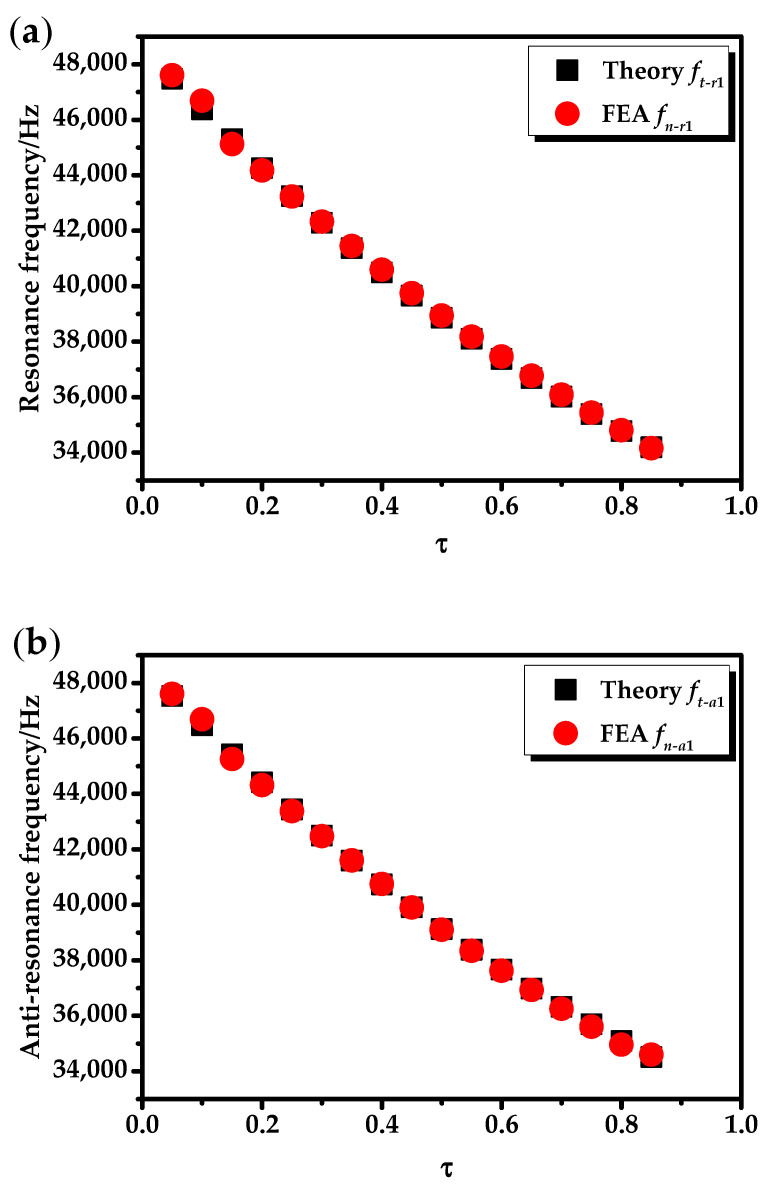
The relationships between the radial resonance, the anti-resonance frequency, and τ at the fundamental mode: (**a**) resonance frequency (*f_r_*_1_); (**b**) anti-resonance frequency (*f_a_*_1_).

**Figure 6:**
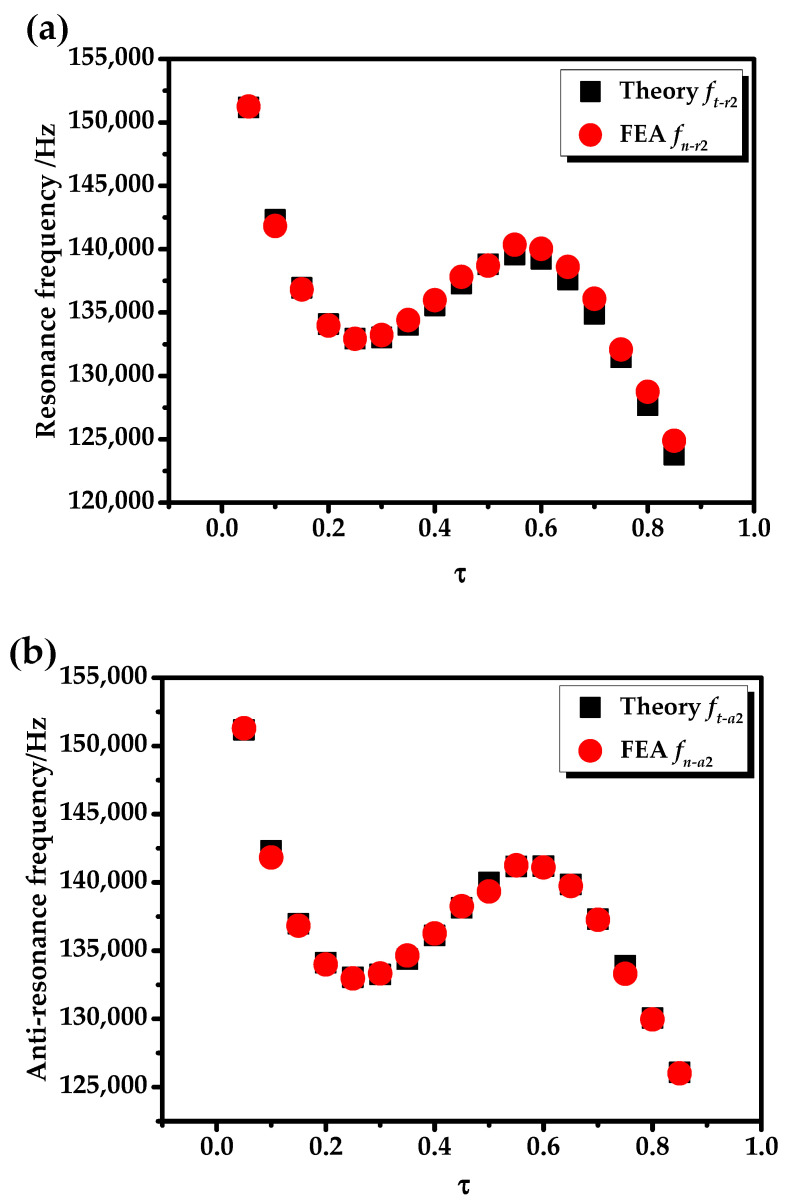
The relationships between the radial resonance, anti-resonance frequency, and τ at the second mode: (**a**) resonance frequency (*f_r_*_2_); (**b**) anti-resonance frequency (*f_a_*_2_).

**Figure 7:**
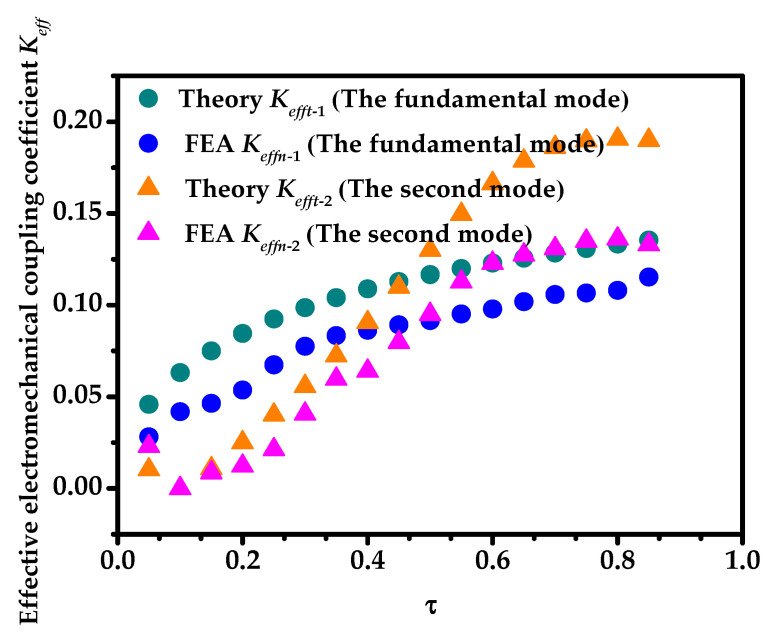
The relationships between the effective electromechanical coupling coefficients and τ at the fundamental mode and the second mode.

Under the no-load state, $Z_{l1}=Z_{l2}=0$. The ratio of the radial geometric dimension of the transducer was defined as τ=(b−a)/(c−a). In the calculation, *b* was gradually increasing and other geometric dimensions were fixed (*a* = 0.010 m, *c* = 0.030 m and *h* = 0.240 m). When the transducer vibrated at the fundamental mode and the second mode, the relationships between the radial resonance (*f_r_*)/the anti-resonance frequency (*f_a_*), the effective electromechanical coupling coefficient (*K_eff_*), and the radial geometric dimension τ were analyzed as shown in Figure 5, Figure 6 and Figure 7. It can be seen from Figure 5, Figure 6 and Figure 7 that the theoretical and simulation results of the transducer are basically consistent.

It can be clearly seen from Figure 5 that as τ increased, the radial resonance (*f_r_*_1_) and the anti-resonance (*f_a_*_1_) frequency of the transducer at the fundamental mode decreased. The reason is that when the overall size of the transducer is unchanged, τ increases, the radial dimension of the piezoelectric ceramic tube increases, the radial dimension of the outer metal tube decreases, and the overall elasticity of the transducer is reduced. It can be seen from Figure 6 that as τ increases, the variation curves of radial resonance frequency (*f_r_*_2_) and the anti-resonance frequency (*f_a_*_2_) at the second mode of the transducer are relatively complex. There are two inflection points in Figure 5 at τ = 0.3 and τ = 0.6.

It can be seen from Figure 7 that when τ increases, the effective electromechanical coupling coefficient (*K_eff_*_1_) at the fundamental mode increases significantly. However, as τ increases, the effective electromechanical coupling coefficient (*K_eff_*_2_) at the second mode decreases first and then increases. When τ is between 0.75 and 0.85, *K_eff_*_2_ is basically unchanged. When τ is around 0.1, *K_eff_*_2_ is almost zero. In the actual engineering design of the transducer, the situation where *K_eff_* is zero should be avoided. The effective electromechanical coupling coefficient should be as large as possible to ensure a higher electromechanical conversion.

As can be seen from Figure 5, Figure 6 and Figure 7, the radial resonance/anti-resonance frequency and the effective electromechanical coupling coefficient of the longitudinally polarized tubular transducer are related to the radial geometry dimension. In the actual design of the transducer, the performance can be improved by changing the radial geometry dimensions.

## 4. Finite Element Analysis of the Radial Composite Tubular Transducer

In this section, COMSOL Multiphysics 5.3a was applied to simulate the vibrational modes of the transducer. The radial relative displacement distribution curves and the modulus of the input electrical impedance can then be obtained. The radial geometric dimensions of the two different radial composite tubular transducers are shown in Table 1. In addition, the ratio of the longitudinal length to the radius of the transducer is defined as $\tau_{1}=h/c$.

**Figure 8:**
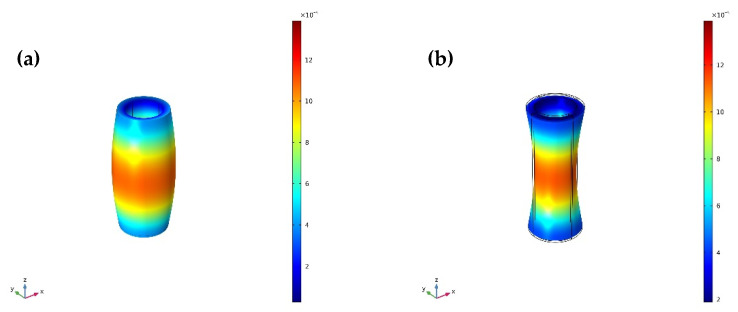
Vibrational mode of the No. 1 transducer with $\tau_{1}=5$ at the fundamental mode (*f_r_*_1_ = 33,997 Hz): (**a**) expansion; (**b**) shrinkage.

**Figure 9:**
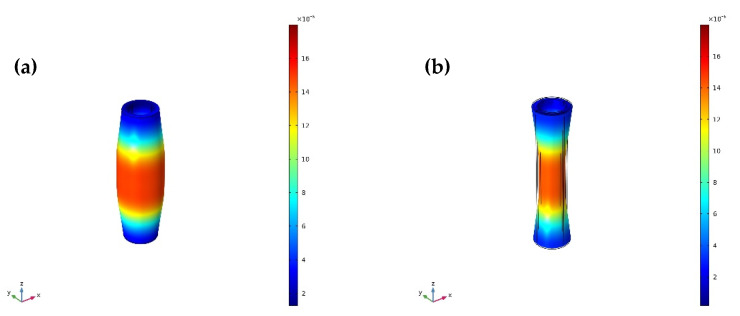
Vibrational mode of the No. 1 transducer with $\tau_{1}=8$ at the fundamental mode (*f_r_*_1_ = 33,541 Hz): (**a**) expansion; (**b**) shrinkage.

**Figure 10:**
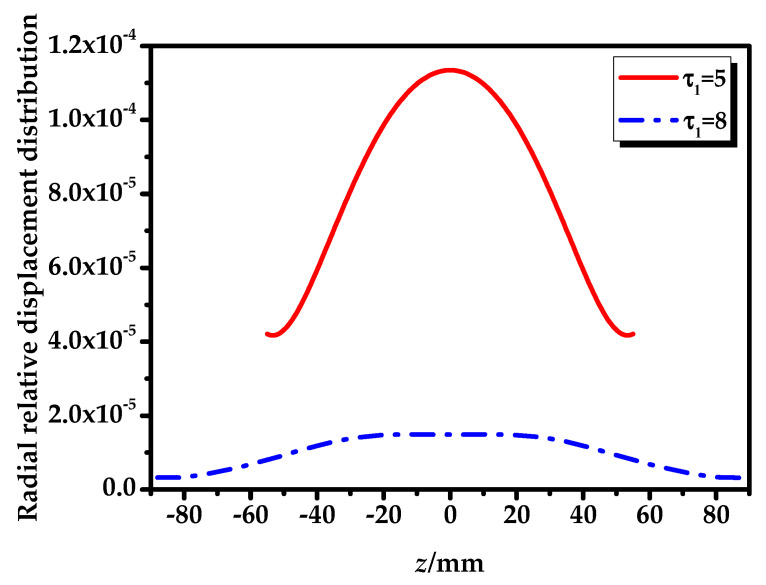
Radial relative displacement distributions of the No. 1 transducer with $\tau_{1}=5,8$ at the fundamental mode.

The vibrational modes and radial relative displacement distribution curves of No. 1 transducer, with $\tau_{1}=5$ and $\tau_{1}=8$ at the fundamental mode are shown in Figure 8, Figure 9 and Figure 10. It can be seen from Figure 8 and Figure 9 that the vibration of the longitudinally polarized radial composite tubular transducer is an axisymmetric radial vibration. It can be seen from Figure 10 that the radial relative displacement distributions along the *z*-axis on the outer surface of the transducers are not completely uniform. Compared with the transducer with $\tau_{1}=5$, the radial relative displacement distribution of the transducer with $\tau_{1}=8$ is more uniform.

**Figure 11:**
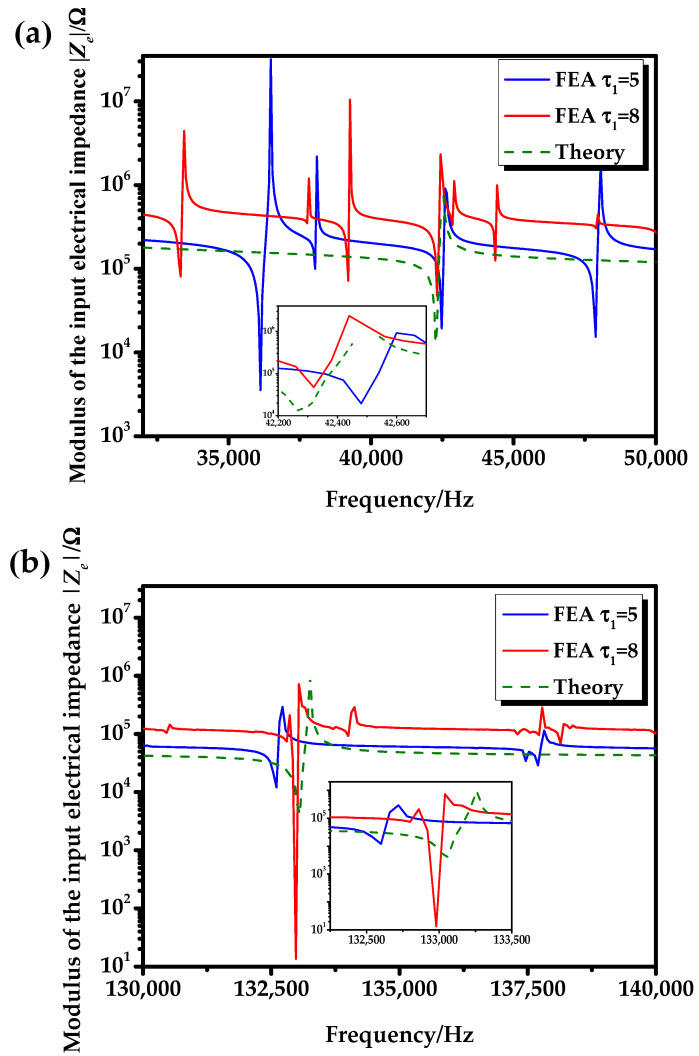
Frequency curves of the input electrical impedance of the No. 2 transducer: (**a**) frequency range is 32–50 kHz; (**b**) frequency range is 130–140 kHz.

The frequency curves of the input electrical impedance of the No. 2 transducer are shown in Figure 11. It can be seen from Figure 11 that the theoretical results and simulation results of the radial vibration of the transducer at the fundamental mode and the second mode are roughly the same. Compared with $\tau_{1}=5$, the frequency curve at $\tau_{1}=8$ is closer to the theoretical result. Compared with the theory, the frequency curve of FEA has multiple vibration modes. The reason for this is that in the process of finite element simulation, multiple modes, such as the longitudinal vibrational mode, radial vibrational mode, and bending vibrational mode of the transducer, were simulated.

The comparisons between the theoretical and simulation results of the No. 1 and No. 2 transducers at the fundamental mode at $\tau_{1}$ = 5, 8 are shown in Table 2 and Table 3. Where *f_t−r_*_1_ and *f_t−a_*_1_ are the theoretical resonance frequency and anti-resonance frequency, respectively; *f_n−r_*_1_ and *f_n−a_*_1_ are the radial resonance frequency and anti-resonance frequency calculated by COMSOL, respectively; and $\Delta 3=\left|f_{n-r1}-f_{t-r1}\right|/f_{n-r1}$ represents the error between the theoretical and simulation results of the resonance frequency at the fundamental mode. $\Delta 4=\left|f_{n-a1}-f_{t-a1}\right|/f_{n-a1}$ represents the error between the theoretical and simulation results of the anti-resonance frequency at the fundamental mode.

As can be seen from Table 2 and Table 3, the analytical theoretical results and COMSOL results are in good agreement. The main reason for the errors (Δ3, Δ4) is that the analytical theory in this paper is based on the plane strain problem, and the vibration of the transducer is regarded as pure radial vibration. Therefore, the analytical theoretical results of the model with an infinite length are inconsistent with the results of the simulation model with a finite length (Figure 10 and Figure 11).

## 5. Conclusions

In this paper, based on the plane strain theory, the equivalent circuits of a longitudinally polarized piezoelectric ceramic long tube and a metal hollow long tube are derived and obtained for the first time. In addition, the electromechanical equivalent circuit of a longitudinally polarized radial composite tubular transducer is obtained. Then, the effects of the radial geometry dimension of the transducer on the vibration characteristics are analyzed. Through the analysis of the research results, the following conclusions can be drawn:

(1) Under the no-load state, when the overall size of the transducer is unchanged, as the proportion of piezoelectric ceramics increases, there are two inflection points on the variation curves of the radial resonance frequency and anti-resonance frequency at the second mode.
(2) The resonance/anti-resonance frequencies of the No. 1 and No. 2 transducers under different ratios of length to radius are obtained using COMSOL. After comparison, the analytical theoretical results and COMSOL results are in good agreement.
(3) In the present work, the vibration characteristics of the transducer at the fundamental mode and the second mode are analyzed. In future work, the higher vibrational order and the effects of the dielectric and load losses on the vibration characteristics will be studied.
(4) The derivation of the electromechanical equivalent circuit of the radial vibration of a longitudinally polarized piezoelectric ceramic tube completes the equivalent circuit theory. The tubular transducer with a large radiation range and power capacity has certain application prospects in the fields of high-power ultrasonic wastewater treatment, ultrasonic degradation, and underwater acoustics.

## Figures and Tables

**Table 1:** Radial geometry dimensions of the two radial composite tubular transducers.

No.	*a*/m	*b*/m	*c*/m
1	0.016	0.020	0.022
2	0.010	0.016	0.030

**Table 2:** Comparisons between the theoretical and simulation results of the No. 1 transducer at $\tau_{1}=5,8$.

$\tau_{1}$	*f_t−r_*_1_/Hz	*f_n−r_*_1_/Hz	Δ3%	*f_t−a_*_1_/Hz	*f_n−a_*_1_/Hz	Δ4%
5	33,286	33,997	2.09	33,465	34,007	1.59
8	33,286	33,541	0.76	33,465	33,583	0.35

**Table 3:** Comparisons between the theoretical and simulation results of the No. 2 transducer at $\tau_{1}=5,8$.

$\tau_{1}$	*f_t−r_*_1_/Hz	*f_n−r_*_1_/Hz	Δ3%	*f_t−a_*_1_/Hz	*f_n−a_*_1_/Hz	Δ4%
5	42,288	42,495	0.49	42,495	42,622	0.30
8	42,288	42,338	0.11	42,495	42,456	0.09

## References

[1] Chilibon I. (2002). Underwater flextensional piezoceramic sandwich transducer. Sens. Actuators A Phys..

[2] Chilibon I. High power ultrasonic piezoceramic vibratory sandwich transducers. Proceedings of the Tenth International Congress on Sound and Vibration.

[3] Loveday P.W. Numerical comparison of patch and sandwich piezoelectric transducers for transmitting ultrasonic waves. Proceedings of the SPIE the International Society for Optical Engineering.

[4] Lin S.Y., Tian H. (2008). Study on the sandwich piezoelectric ceramic ultrasonic transducer in thickness vibration. Smart Mater. Struct..

[5] Wang J.J., Qin L., Song W.B., Shi Z., Song G. (2018). Electromechanical Characteristics of Radially Layered Piezoceramic/Epoxy Cylindrical Composite Transducers: Theoretical Solution, Numerical Simulation, and Experimental Verification. IEEE Trans. Ultrason. Ferroelectr. Freq. Control.

[6] Kim J.O., Hwang K.K., Jeong H.G. (2004). Radial vibration characteristics of piezoelectric cylindrical transducers. J. Sound Vib..

[7] Li G., Gong J., Wang T., Qiu C., Xu Z. (2018). Study on the broadband piezoelectric ceramic transducer based on radial enhanced composite structure. Ceram. Int..

[8] Jia L.Y., Zhang G.B., Zhang X.F., Yao Y., Lin S. (2017). Study on tangentially polarized composite cylindrical piezoelectric transducer with high electro-mechanical coupling coefficient. Ultrasonics.

[9] Iula A., Lamberti N., Carotenuto R., Pappalardo M. (1999). Analysis of the radial symmetrical modes of thin piezoceramic rings. IEEE Trans. Ultrason. Ferroelectr. Freq. Control.

[10] Iula A., Lamberti N., Pappalardo M. (1996). A model for the theoretical characterization of thin piezoceramic rings. IEEE Trans. Ultrason. Ferroelectr. Freq. Control.

[11] Ganilova O., Lucas M., Cardoni A. (2011). Analytical model of the cymbal transducer dynamics, radial vibration of the piezoelectric disc. Proc. Inst. Mech. Eng. Part C J. Mech. Eng. Sci..

[12] Guo N., Cawley P., Hitchings D. (1992). The finite element analysis of the vibration characteristics of piezoelectric discs. J. Sound Vib..

[13] Lin S.Y., Hu J., Fu Z.Q. (2013). Electromechanical characteristics of piezoelectric ceramic transformers in radial vibration composed of concentric piezoelectric ceramic disk and ring. Smart Mater. Struct..

[14] Lin S.Y. (2006). Study on a new type of radial composite piezoelectric ultrasonic transducers in radial vibration. IEEE Trans. Ultrason. Ferroelectr. Freq. Control.

[15] Lin S.Y. (2007). Electro-mechanical equivalent circuit of a piezoelectric ceramic thin circular ring in radial vibration. Sens. Actuators A Phys..

[16] Lin S.Y. (2004). Study on the equivalent circuit and coupled vibration for the longitudinally polarized piezoelectric ceramic hollow cylinders. J. Sound Vib..

[17] Aronov B. (2009). Coupled vibration analysis of the thin-walled cylindrical piezoelectric ceramic transducers. J. Acoust. Soc. Am..

[18] Hongwei W. (2016). Finite element analysis and testing of the stacked piezoelectric composite ring array transducer. J. Funct. Mater..

[19] Tolliver L., Xu T.B., Jiang X. (2013). Finite element analysis of the piezoelectric stacked-HYBATS transducer. Smart Mater. Struct..

[20] Nantawatana W., Seemann W. (2008). Dynamic response analysis of the two stators hybrid transducer type piezoelectric ultrasonic motor using finite element simulation. Proc. Appl. Math. Mech..

[21] Lin S.Y., Wang S.J., Fu Z.Q. (2012). Electro-mechanical equivalent circuit for the radial vibration of the radially poled piezoelectric ceramic long tubes with arbitrary wall thickness. Sens. Actuators A Phys..

[22] Tsysar S.A., Sinelnikov Y.D., Sapozhnikov O.A. (2011). Characterization of cylindrical ultrasonic transducers using acoustic holography. Acoust. Phys..

[23] Aronov B. (2005). The energy method for analyzing the piezoelectric electroacoustic transducers. J. Acoust. Soc. Am..

[24] Huang Y.H., Ma C.C. (2012). Experimental measurements and finite element analysis of the coupled vibrational characteristics of piezoelectric shells. IEEE Trans. Ultrason. Ferroelectr. Freq. Control.

[25] Grigorenko A.Y., Loza I.A. (2012). Solution of the problem of nonaxisymmetric free vibrations of piezoceramic hollow cylinders with axial polarization. J. Math. Sci..

[26] Mason W.P. (1948). Electro-Mechanical Transducers and Wave Filters.

[27] Martin G.E. (1963). Vibrations of Longitudinally Polarized Ferroelectric Cylindrical Tubes. J. Acoust. Soc. Am..

[28] Bybi A., Mouhat O., Garoum M., Drissi H., Grondel S. (2019). One-dimensional equivalent circuit for ultrasonic transducer arrays. Appl. Acoust..

[29] Silva T.M., Clementino M.A., Erturk A., De Marqui C. (2018). Equivalent electrical circuit framework for nonlinear and high-quality factor piezoelectric structures. Mechatronics.

[30] Feng F.L., Shen J.Z., Deng J.J. (2007). A 2D equivalent circuit of piezoelectric ceramic ring for transducer design. Ultrasonics.

[31] Jalili H., Goudarzi H. (2009). Modeling the hollow cylindrical piezo-ceramics with axial polarization using equivalent electro-mechanical admittance matrix. Sens. Actuators A Phys..

[32] Luan J.D., Zhang J.Q., Wang R.Q. (2005). Piezoelectric Transducers and Arrays.

[33] Liu S.Q., Lin S.Y. (2009). The analysis of the electro-mechanical model of the cylindrical radial composite piezoelectric ceramic transducer. Sens. Actuators A Phys..

